# Epidermal Tissue Underlying an Exposed Skull Fixation Plate: A Case Report

**DOI:** 10.7759/cureus.94958

**Published:** 2025-10-20

**Authors:** Shuya Kurono, Kentaro Wada, Tsuneo Yasumura, Otone Endo, Takeshi Okada

**Affiliations:** 1 Department of Neurosurgery, The Aichi Prefectural Federation of Agricultural Cooperatives for Health and Welfare Kainan Hospital, Yatomi, JPN; 2 Department of Plastic Surgery, The Aichi Prefectural Federation of Agricultural Cooperatives for Health and Welfare Kainan Hospital, Yatomi, JPN; 3 Department of Neurological Surgery, The Aichi Prefectural Federation of Agricultural Cooperatives for Health and Welfare Kainan Hospital, Yatomi, JPN

**Keywords:** case report, clamp-type titanium plate, craniotomy, neurosurgery, plate exposure

## Abstract

For bone flap fixation after craniotomy, clamp-type titanium plates are widely used because of their procedural simplicity. However, mechanical irritation caused by the thickness of these plates is known to result in scalp thinning and plate exposure as complications. We report a rare case in which full-thickness skin, including hair, was found beneath an exposed clamp-type titanium plate. The patient was a woman in her 60s who presented with an exposed plate in the frontal region approximately 20 years after a craniotomy. During surgical removal of the plate, epithelial-like tissue with hair was observed directly underneath the outer clamp component. Pathological examination confirmed a full-thickness skin layer composed of epidermis, dermis, subcutaneous fatty tissue, and appendages such as hair follicles and glands. Signs of localized inflammation were also present. Potential mechanisms for this epithelial entrapment include intraoperative inclusion of a free skin fragment or secondary migration of skin appendages caused by chronic irritation. The accumulation of secretions and desquamated debris from such epithelium beneath a solid plate can lead to inflammation and infection. In this case, the exposed plate was removed, the underlying tissue was debrided, and the wound was closed. The patient has been followed for two years postoperatively without any complications. Although this is an unusual case, given the risk of epithelial tissue forming beneath the plate, removal of the plate and debridement of the underlying tissue should be considered when a clamp-type plate becomes exposed.

## Introduction

Various methods are used to fix the bone flap in the desired position during closure after a craniotomy. While wire sutures were used in the past, titanium plates have become common, and ceramic and bioabsorbable plates have been introduced more recently [1]. However, because of their high fixation strength and low bioreactivity, titanium plates are still used in many craniotomy procedures. Clamp-type plates, in particular, are valued for their procedural simplicity [2].

A known complication of titanium plate use is mechanical irritation of adjacent tissues, which can lead to thinning of the overlying scalp and subsequent plate exposure [3]. Management of exposed plates varies and may include plate removal or coverage with local flaps.

We report a case in which full-thickness skin, including hair, was pathologically identified beneath an exposed clamp-type titanium plate. The pathological findings are presented, and appropriate treatment considerations for such cases of plate exposure are discussed.

## Case presentation

**Figure 1:**
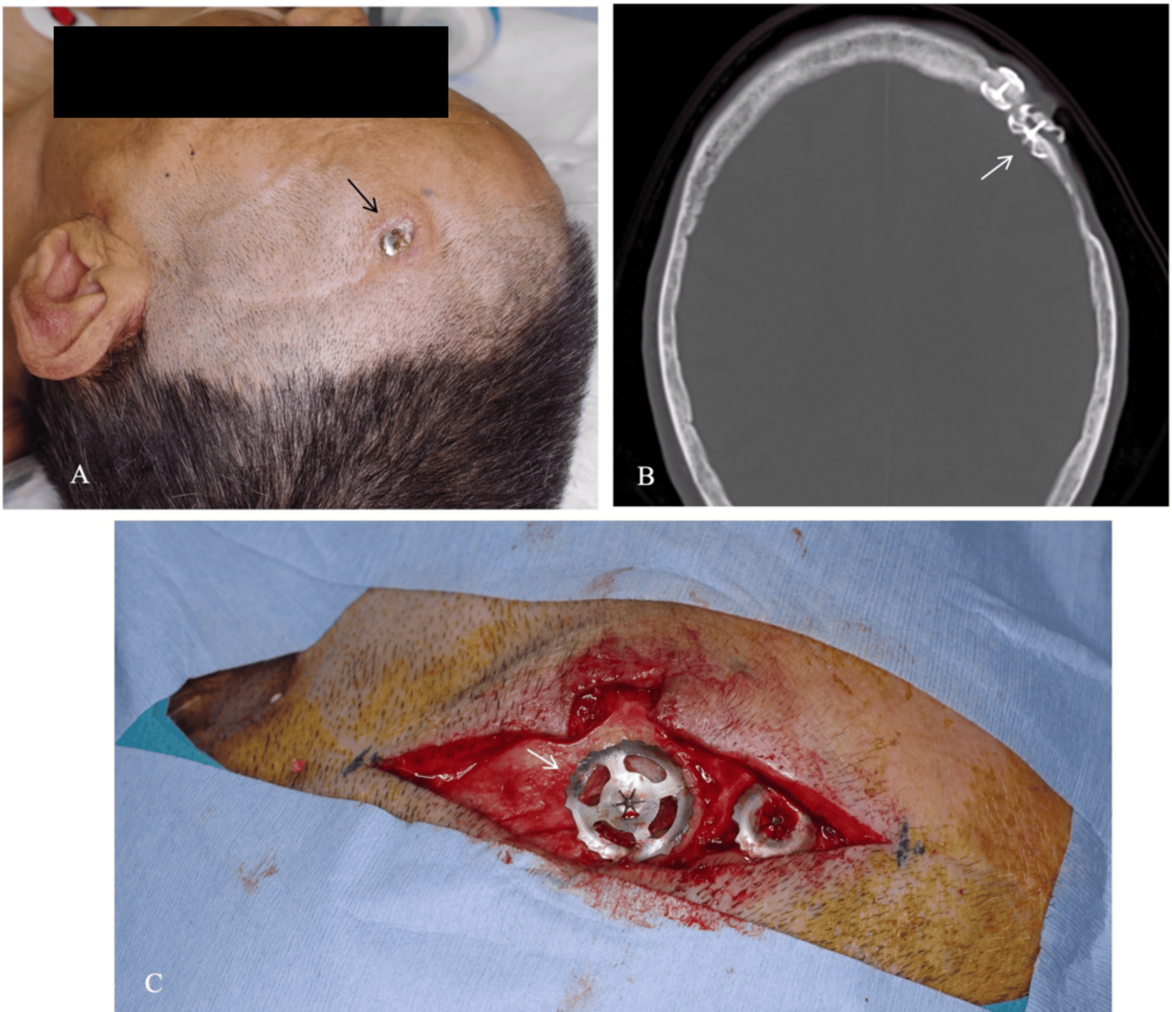
(A) A metallic plate (arrow) is exposed in the left frontal region. (B) CT of the head shows that the exposed hardware at the same site represents a clamp-type metallic plate (arrow). (C) After scalp incision and bone exposure, a metallic plate (arrow), identified as a CranioFix® (B. Braun Aesculap Co., Tokyo, Japan), is confirmed.

The patient was a woman in her 60s with a history of left frontotemporal craniotomy for clipping of a ruptured left middle cerebral artery aneurysm. Approximately 20 years after the procedure, she noticed exposure of a metallic plate in the frontal region (Figure 1A). After monitoring the condition for about two years, she presented to our hospital because of the gradual enlargement of the exposed area. CT confirmed that the exposed plate was a clamp-type system (Figure 1B). Although no signs of infection were identified, progressive exposure warranted surgical intervention for plate removal and wound closure.

**Figure 2:**
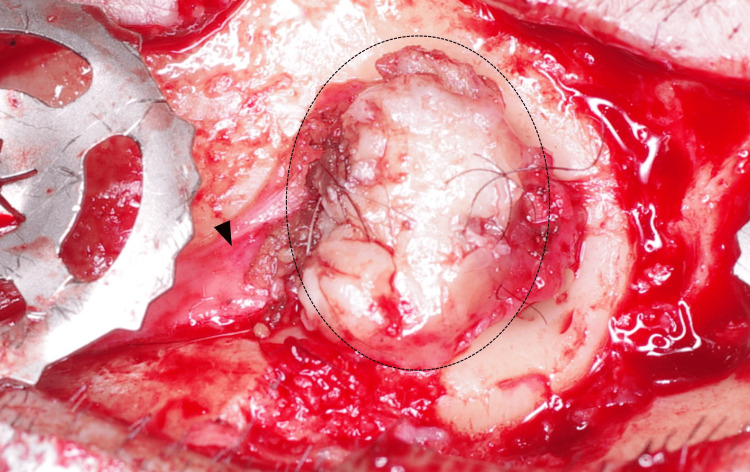
Epithelium-like tissue (within the dotted line) is observed after removal of the plate (left). Hair shafts can be seen growing from the tissue. Beneath this layer, granulation-like tissue (arrowhead) is also visible. The difference in color clearly distinguishes the two tissue types.

Under general anesthesia, an incision was made along the previous scar line to expose the plate (Figure 1C). The plate was identified as a CranioFix® 2 clamp-type plate (FF491T; B. Braun Aesculap Co., Tokyo, Japan). After removal of the outer clamp component, epithelium-like tissue with hair was observed directly beneath it (Figure 2). Beneath this epithelial-like layer, granulation-like tissue was also identified (Figure 2), indicating the presence of two distinct tissue types. These tissues were excised for pathological examination. After removal of the epithelium-like tissue, the skin was sutured, and the surgery was completed. The postoperative course was uneventful, with no wound dehiscence or infection. The staples were removed on postoperative day 7, and the patient was discharged home on postoperative day 10.

**Figure 3:**
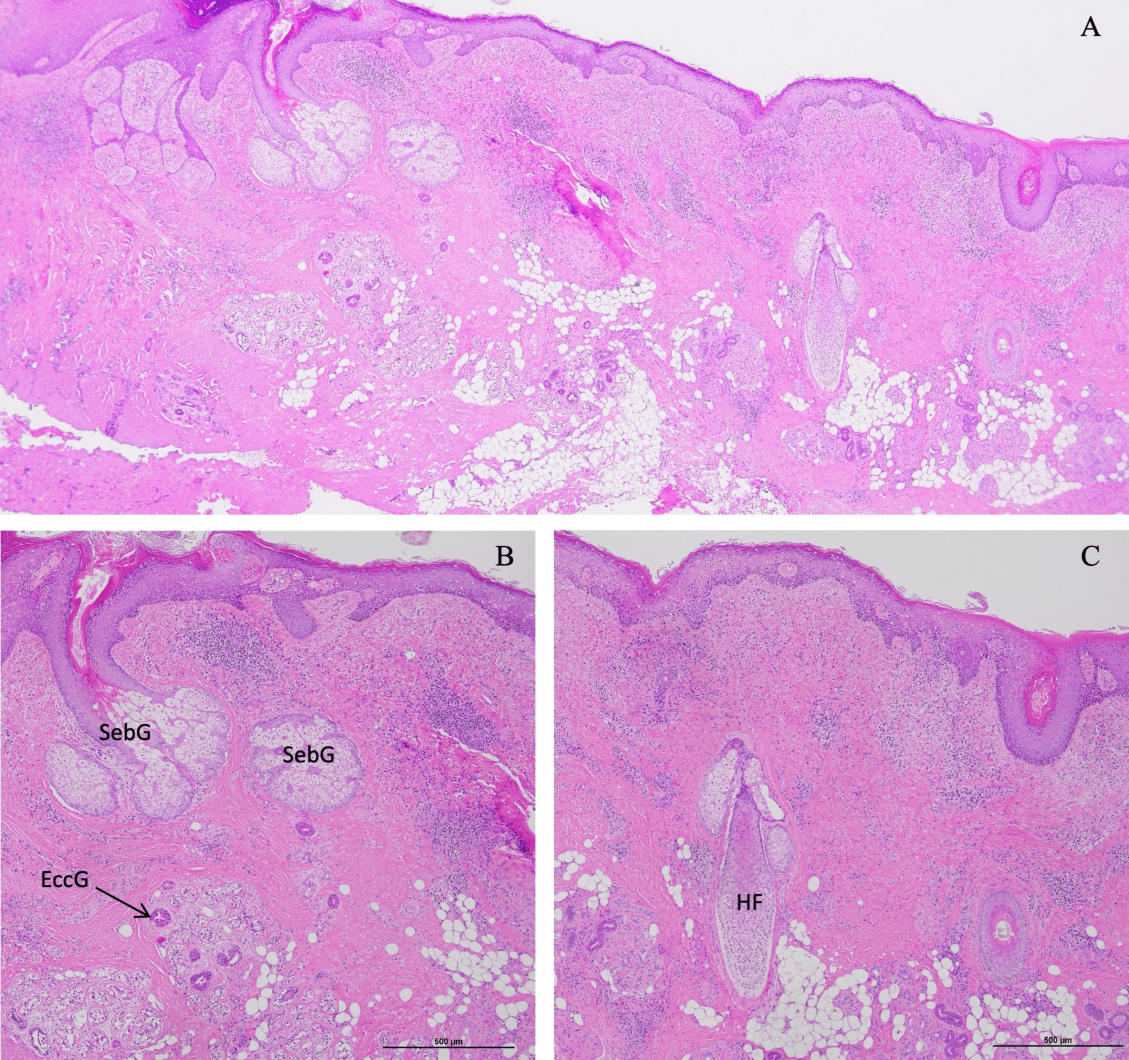
Histopathological findings of the excised epithelial-like tissue. (A) H&E stain (×20 magnification). A full-thickness skin layer composed of epidermis, dermis, and subcutaneous fatty tissue is observed. (B, C) H&E stain (×40 magnification). Skin appendages are also present, including an EccG, a SebG, and an HF. EccG, eccrine glands; HF, hair follicle; SebG, sebaceous gland

Pathological examination of the excised tissue confirmed the presence of a full-thickness skin layer composed of epidermis, dermis, and subcutaneous fatty tissue (Figure 3). Skin appendages, including sweat glands, sebaceous glands, and hair follicles, were also observed. Histologically, this tissue was indistinguishable from normal skin. The dermis showed infiltration of lymphocytes and plasma cells, primarily around small blood vessels, indicating localized inflammation. More than two years after wound closure, the patient remains well, with no wound dehiscence, infection, or bone flap instability.

## Discussion

Titanium plates are widely used for bone flap fixation in craniotomy because of their high fixation strength and low bioreactivity [4]. Among these, clamp-type titanium plates are popular for their ease of use and strong fixation [5]. However, the thickness of such plates can cause mechanical irritation to the overlying skin and subcutaneous tissue, often leading to plate exposure [6]. Typically, in cases of long-standing plate exposure or infection, the gap between the plate and bone flap becomes filled with osseous or granulation tissue upon plate removal. In our case, however, we found skin tissue with all its appendages, including hair, directly beneath the outer plate. A similar case of epithelium-like tissue found beneath a mesh plate used for cranioplasty has been reported [7], but to the best of our knowledge, no reports have described such tissue under a solid metal plate as in our case.

We propose three possible mechanisms for the entrapment of epithelial tissue beneath the plate, taking into account the characteristics of clamp-type systems.

The first mechanism involves intraoperative entrapment of a free fragment of epithelial tissue under the plate during closure. The design of clamp-type plates, particularly when placed near the wound edge, may increase the risk of catching and securing epithelial tissue underneath.

The second mechanism is similar, involving the inadvertent suturing of epithelial tissue into the wound during closure, followed by the subsequent migration of this tissue through a plate hole to settle beneath it. The difference from the first mechanism lies in the timing of epithelial entrapment, whether the free fragment is present from the start or whether the sutured tissue migrates later. Dermoid cysts, for instance, are known to arise not only from embryonic entrapment of ectodermal elements but also from post-traumatic implantation of epithelial tissue into subcutaneous layers [8,9]. In both of these mechanisms, the entrapped epithelial tissue could later contribute to wound infection or plate exposure through desquamation and accumulation of secretions.

A third mechanism involves mechanical irritation caused by the metal plate, which can lead to thinning of the overlying skin. Subsequently, skin appendages adjacent to a plate hole could migrate underneath and become established. In this scenario, wound dehiscence would not result from epithelial desquamation or secretion but rather from mechanical irritation and stress concentration created by the plate and its holes.

Zhang et al. reported a similar case of epithelial-like tissue found beneath a mesh plate used for cranioplasty [7]. They proposed that this phenomenon, termed “dermointegration,” occurs when thinned tissue migrates through mesh holes, adheres to the dural surface, and proliferates to form a continuous epithelial layer. They suggested that this epithelial barrier might provide some resistance to foreign body infection, allowing conservative management in patients unsuitable for general anesthesia. A similar phenomenon was observed in our case with a clamp-type plate. However, unlike a mesh plate, where secretions and desquamated cells from the underlying epithelium can be cleared through mesh pores, a clamp-type plate with an enclosed space beneath it is more likely to retain such materials [10]. This retention could lead to a buildup of secretions and debris, similar to an epidermoid cyst within a hair follicle, increasing the risk of inflammation and infection. In our case, the pathological findings indeed demonstrated localized inflammation (Table 1).

**Table 1:** Comparison between dermointegration (Zhang et al.’s case) and the present case

Parameter	Dermointegration (Zhang et al.’s case)	Present case
Causative device	Mesh plate	Clamp-type plate
Mechanism of tissue migration	Thinned tissue migrates through mesh holes and reproliferates on the dural surface	Skin appendages migrate underneath from areas adjacent to a plate hole and become established
Drainage of secretions and debris	Can potentially be cleared through mesh pores	More likely to be retained in the space beneath the plate
Clinical outcome/risk	May offer some resistance to foreign body infection	Increased risk of inflammation and infection (pathological findings showed localized inflammation)

In this case, plate exposure was observed 20 years postoperatively, a relatively long interval. Preoperative examination of the exposed plate revealed that, although it was located near the wound margin, the original skin incision line remained intact. This finding suggests that mechanical irritation from the plate was the primary factor contributing to plate exposure, making the third proposed mechanism the most plausible.

For treatment, we initially considered covering the plate with a local flap without removal. However, that approach would have carried the risk of burying pre-existing epithelial tissue beneath the flap. Therefore, removal of the plate and curettage of the underlying pathological tissue were essential before flap placement in this case. Our approach of removing the outer plate and closing the wound achieved a favorable outcome. We also emphasize that, at two years postoperatively, there has been no recurrence of plate exposure.

## Conclusions

We report a case of epithelial tissue ingrowth beneath a clamp-type plate following postoperative exposure. The accumulation of secretions and desquamated debris from this epithelium beneath the plate poses a risk of inflammation and infection. As demonstrated in this case, epidermal tissue can persist directly under the plate; therefore, when such findings are observed, treatment should include removal of the plate and debridement of the underlying pathological tissue.
